# Subtype C gp140 Vaccine Boosts Immune Responses Primed by the South African AIDS Vaccine Initiative DNA-C2 and MVA-C HIV Vaccines after More than a 2-Year Gap

**DOI:** 10.1128/CVI.00717-15

**Published:** 2016-06-06

**Authors:** Glenda E. Gray, Kenneth H. Mayer, Marnie L. Elizaga, Linda-Gail Bekker, Mary Allen, Lynn Morris, David Montefiori, Stephen C. De Rosa, Alicia Sato, Niya Gu, Georgia D. Tomaras, Timothy Tucker, Susan W. Barnett, Nonhlanhla N. Mkhize, Xiaoying Shen, Katrina Downing, Carolyn Williamson, Michael Pensiero, Lawrence Corey, Anna-Lise Williamson

**Affiliations:** a Perinatal HIV Research Unit, Faculty of Health Sciences, University of the Witwatersrand, Johannesburg, South Africa; b South African Medical Research Council, Cape Town, South Africa; c Fenway Health and Beth Israel Deaconess Medical Center, Boston, Massachusetts, USA; d Fred Hutchinson Cancer Research Center, Seattle, Washington, USA; e Institute of Infectious Disease and Molecular Medicine, University of Cape Town, Cape Town, South Africa; f DAIDS, NIAID, NIH, Washington, DC, USA; g National Institute for Communicable Diseases of the National Health Laboratory Services, Johannesburg, South Africa; h Duke University, Durham, North Carolina, USA; i Novartis Vaccines, Cambridge, Massachusetts, USA; j National Health Laboratory Service, Cape Town, South Africa; National Institute of Allergy and Infectious Diseases

## Abstract

A phase I safety and immunogenicity study investigated South African AIDS Vaccine Initiative (SAAVI) HIV-1 subtype C (HIV-1C) DNA vaccine encoding Gag-RT-Tat-Nef and gp150, boosted with modified vaccinia Ankara (MVA) expressing matched antigens. Following the finding of partial protective efficacy in the RV144 HIV vaccine efficacy trial, a protein boost with HIV-1 subtype C V2-deleted gp140 with MF59 was added to the regimen. A total of 48 participants (12 U.S. participants and 36 Republic of South Africa [RSA] participants) were randomized to receive 3 intramuscular (i.m.) doses of SAAVI DNA-C2 of 4 mg (months 0, 1, and 2) and 2 i.m. doses of SAAVI MVA-C of 1.45 × 10^9^ PFU (months 4 and 5) (*n* = 40) or of a placebo (*n* = 8). Approximately 2 years after vaccination, 27 participants were rerandomized to receive gp140/MF59 at 100 μg or placebo, as 2 i.m. injections, 3 months apart. The vaccine regimen was safe and well tolerated. After the DNA-MVA regimen, CD4^+^ T-cell and CD8^+^ T-cell responses occurred in 74% and 32% of the participants, respectively. The protein boost increased CD4^+^ T-cell responses to 87% of the subjects. All participants developed tier 1 HIV-1C neutralizing antibody responses as well as durable Env binding antibodies that recognized linear V3 and C5 peptides. The HIV-1 subtype C DNA-MVA vaccine regimen showed promising cellular immunogenicity. Boosting with gp140/MF59 enhanced levels of binding and neutralizing antibodies as well as CD4^+^ T-cell responses to HIV-1 envelope. (This study has been registered at ClinicalTrials.gov under registration no. NCT00574600 and NCT01423825.)

## INTRODUCTION

In response to a devastating HIV-1 subtype C epidemic in southern Africa, the South African AIDS Vaccine Initiative (SAAVI), a lead program of the South African Medical Research Council (SAMRC), in collaboration with the University of Cape Town (UCT) and the U.S. National Institutes of Health, developed a subtype C HIV (HIV-1C) vaccine regimen consisting of two multigene recombinant vaccines—a DNA vaccine and an MVA vaccine—expressing matched HIV-1C proteins (1). The HIV-1C gene insertions were selected from representative circulating viral isolates in South Africa (2, 3). Preclinical immunogenicity studies performed without the HIV-1 C protein boost in both mice (4) and baboons demonstrated that the DNA/MVA regimen elicited potent T-cell lymphocyte responses as well as binding antibody responses to HIV-1C gp120 (5).

This first-in-human study using the SAAVI DNA-C2 and SAAVI MVA-C vaccines evaluated the safety and immunogenicity of the DNA/MVA prime-boost regimen in both the Republic of South Africa (RSA) and the United States (HVTN [HIV Vaccine Trials Network] 073/SAAVI 102). In an attempt to improve HIV-specific antibody responses, a V2-deleted envelope subunit HIV-1C protein vaccine adjuvanted with MF59 was used as an additional boost (HVTN 073E/SAAVI 102E), based on recent promising preclinical and clinical immunogenicity studies (6). We investigated the effect of the protein boost on both cellular and humoral immunity.

## MATERIALS AND METHODS

### Study design.

HVTN 073/SAAVI 102, a phase I randomized, double-blind placebo-controlled trial designed to evaluate the safety and immunogenicity of the SAAVI DNA-C2 and SAAVI MVA-C vaccines (Table 1 and Table 2), was conducted in non-HIV-infected healthy vaccinia virus-naive adult participants at two RSA sites (Perinatal HIV Research Unit, Soweto, South Africa, and the Desmond Tutu HIV Centre, Cape Town, South Africa) and two U.S. sites (Brigham & Women's Hospital, Boston, MA, and Fenway Health, Boston, MA). The trial design is shown in Table 1 and was extended to evaluate a subtype C V2-deleted gp140 vaccine with MF59 adjuvant (Table 2) after the results of the RV144 study indicated that the addition of a protein boost could enhance viral-vector-mediated immunogenicity.

**TABLE 1:** Trial schema for initial DNA/MVA regimen (HVTN 073)

No. of participants (*n* = 48)	Treatment arm	Dose		Regimen administered the indicated no. of mos (days) after the first injection				
		DNA-C2 (mg)	MVA-C (PFU)	0 (0)	1 (28)	2 (56)	4 (112)	5 (140)
40	T1	4	1.45 × 10^9^	DNA-C2	DNA-C2	DNA-C2	MVA-C	MVA-C
8	C1	0	0	Placebo	Placebo	Placebo	Placebo	Placebo

**TABLE 2:** Trial schema for study extension (HVTN 073E)

No. of participants (*n* = 27)	Previous regimen^*a*^	Treatment group	Subtype C gp140/MF59 dose (μg)	Regimen administered the indicated no. of mos (days) after first extension injection	
				0 (0)	3 (84)
16	DDDMM	T1/T2	100	gp140/MF59	gp140/MF59
6	DDDMM	T1/C2	0	Placebo	Placebo
1	CCCC	C1/T2	100	gp140/MF59	gp140/MF59
4	CCCC	C1/C2	0	Placebo	Placebo

a D, DNA-C2; M, MVA-C; C, placebo.

### Vaccines.

SAAVI DNA-C2 consisted of two DNA plasmids, pVRCgrttnC (expressing HIV-1C Gag-reverse transcriptase-Tat-Nef [*grttnC*] polyprotein from the Du422 isolate) and pVRCgp150CT (expressing an HIV-1C truncated Env from the Du151 isolate [3]), manufactured by Althea Technologies, Inc. (San Diego, CA, USA) and mixed in equal weights (1:1 [wt/wt]) for the vaccine. SAAVI MVA-C contained *grttnC* under the control of the vaccinia virus 40K promoter inserted into the Del III region, and *gp150CT*, under the control of the vaccinia virus 13 promoter inserted into the 49/50 region (5). The SAAVI MVA-C vaccine was manufactured by Therion Biologics Corporation (defunct; Cambridge, MA, USA) (5). The Novartis HIV-1C gp140 vaccine (manufactured in Emeryville, CA, USA) was a recombinant oligomeric V2-deleted gp140 vaccine (gp140ΔV2.TV1) produced in Chinese hamster ovary cells. The gp140 vaccine was derived from a South African subtype C primary isolate, TV1, and was given with MF59 (6). The placebo was 0.9% sodium chloride (for injection).

### Study population.

Participants were volunteers aged 18 to 45 years and were classified as healthy on the basis of medical history, physical examination, laboratory tests, troponin levels, and electrocardiograms. Participants were at low risk for HIV infection, according to risk assessment and risk criteria developed by each site. Participants were randomized in a 5:1 ratio to the treatment group or placebo control group (Table 1). For the study extension, consenting eligible participants were rerandomized in a 2:1 ratio to receive the subtype C gp140/MF59 vaccine or the placebo, given twice, on dates 3 months apart, around 2 years (median, 2.3 years; range, 2.0 to 2.4 years) after completion of the initial regimen (Table 2).

### Safety assessment.

Safety evaluations included physical examinations, standard serum chemistry and hematological tests, cardiac troponin T tests, and 12 lead electrocardiogram (EKG) tests to identify potential cardiac adverse events (AEs) after receipt of MVA.

Reactogenicity symptoms were assessed for 3 days following each vaccination until resolution. AEs were recorded for each participant for the 12 months of the original study and for 15 months in the study extension. Reactions and AEs were graded as mild, moderate, or severe according to standard criteria (http://rcc.tech-res.com/safetyandpharmacovigilance/). Risk reduction counseling was provided at each visit.

### Immunogenicity assessment.

Immunogenicity endpoints for both humoral and cellular responses were measured at the primary immunogenicity time points, 2 weeks after each MVA-C/placebo and each protein/placebo vaccination, i.e., after the fourth, fifth, sixth, and seventh vaccinations, and at the durability time point, which was 6 months after the final MVA/placebo or protein/placebo (fifth and seventh) vaccinations. Endpoint assays for assessing the humoral responses included evaluation of binding of antibodies to Env and Gag by enzyme-linked immunosorbent assay (ELISA) and binding antibody multiplex assay (BAMA) and of HIV-1-specific neutralizing antibodies. The endpoint assay for assessing cellular responses was flow cytometry with intracellular cytokine staining (ICS) for gamma interferon (IFN-γ), interleukin-2 (IL-2), and tumor necrosis alpha (TNF-α).

### ICS assay.

Flow cytometry was used to examine HIV-1-specific CD4^+^ and CD8^+^ T-cell responses (7, 8). Peripheral blood mononuclear cells (PBMC) were isolated and cryopreserved within 8 h of venipuncture from sodium heparin-anticoagulated blood obtained at the primary immunogenicity time points (9). PBMC were stimulated with peptide pools of HIV-1 global potential T-cell epitopes (PTEG) (10) that spanned the proteins encoded by the vaccine construct.

### Binding antibodies.

Binding of antibodies to protein antigens was assessed both by validated ELISA and by binding antibody multiplex assays (BAMA). Serum dilutions of 1:20 were used for ELISAs to analyze ConS gp140 and p55 Gag for participants in the main study and to analyze ConS, Du151.2 gp140, and gp140ΔV2.TV1 for participants in the study extension. Samples that were saturated at this dilution were further diluted to 1:2,000 (11).

Serum HIV-1-specific IgG responses (1:20 or 1:50) were also evaluated by BAMA against ConS gp140 CFI, gp41 Env, p24 Gag, and gp140ΔV2.TV1 at baseline, at the primary immunogenicity time points, just prior to the first protein/placebo immunization, and 6 months after the final boost (11, 12). Antibody titers (expressed as the area under the dilution curve [AUC]) were calculated from serum dilutions (1.50 to 1:388,800) at a given visit.

Linear epitope mapping was evaluated on a subset of vaccines by peptide microarray with 15-mer peptides overlapping by 12, covering consensus Env strains (gp160) and vaccine strains (gp120), as previously described (13, 14, 15).

### Neutralizing antibody assay.

HIV-1-specific neutralizing antibody assays were performed at baseline and at the primary immunogenicity time points. Neutralizing antibodies were measured as a function of reductions in Tat-regulated luciferase reporter gene expression after a single round of infection in TZM-bl cells against tier 1 and tier 2 HIV-1 isolates (16).

### Statistical methods.

All data from enrolled participants who received at least one vaccination were analyzed using SAS and R statistical software.

For ICS, two-by-two contingency tables comparing the HIV-1 peptides (stimulated and negative control for each peptide pool) for the two T-cell subsets (CD4^+^ and CD8^+^) expressing IFN-γ and/or IL-2 were constructed. A one-sided Fisher's exact test applied to each table tested whether the number of cytokine-producing cells for the stimulated data was equal to that for the negative-control data. Since multiple individual tests (for each peptide pool) were conducted simultaneously, a multiplicity adjustment was made to the individual peptide pool *P* values using the Bonferroni-Holm adjustment method (17). The adjusted *P* values were used to determine positivity, with values of ≤0.00001 indicating a positive response. If one peptide pool for a specific gene was positive, then the overall response to the gene was considered positive. If any peptide pool was positive for a T-cell subset, then the overall response rate for that T-cell subset was considered positive. For the ICS, two-sided 95% confidence intervals were calculated using the score test method of Agresti and Coull (18).

For the ELISA response, a response to a peptide was considered positive if the difference in duplicate antigen-containing and non-antigen-containing wells corresponded to an optical density (OD) of >0.2 and the OD was ≥3 times the day 0 (baseline) OD.

For the BAMA, postenrollment samples were considered positive if they met three conditions: (i) the mean fluorescence intensity (MFI) value minus the blank value was greater than or equal to the antigen-specific cutoff value (based on averages + 3 standard deviations of results from 60 seronegative plasma samples); (ii) the MFI value minus the blank value was greater than 3 times the baseline (day 0) MFI value minus the blank value; and (iii) the MFI value was greater than 3 times the baseline MFI value. The values corresponding to the MFI minus the blank responses were used to summarize the magnitude at a given time point.

For neutralizing antibodies, a response to an isolate was considered positive if the titer was ≥10, where a titer was defined as the sample dilution that reduces infection by half relative to the results seen with untreated virus or with virus treated with prevaccination serum.

## RESULTS

### Participant accrual, demographics, and vaccine safety.

**FIG 1:**
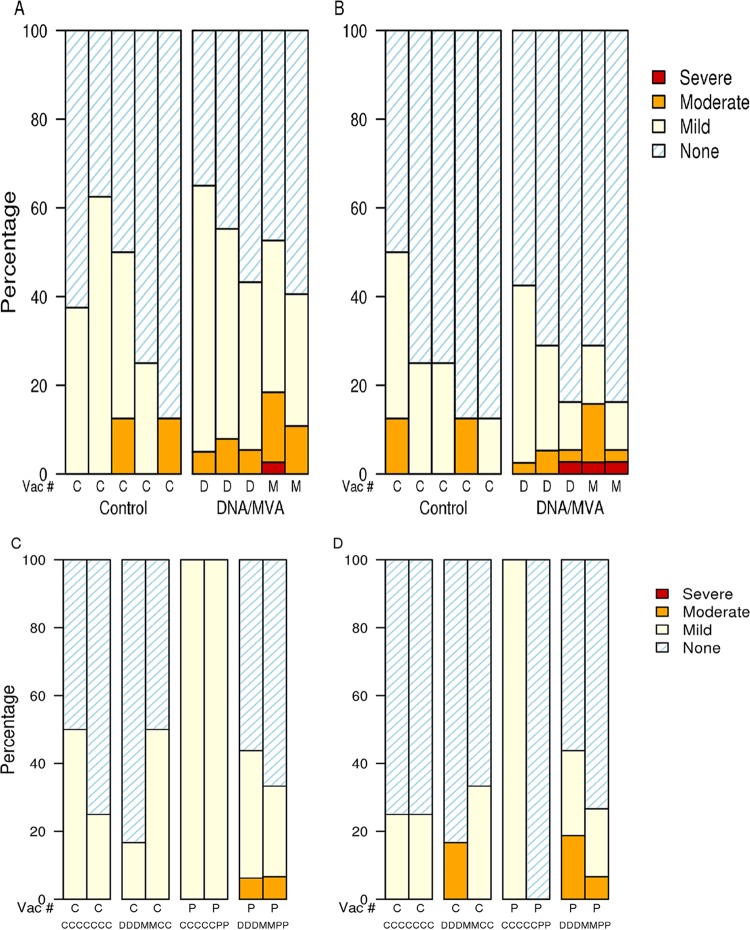
Injection site and systemic reactogenicity. The severity of reactogenicity symptoms is shown for the DNA/MVA prime-boost study (panels A and B) and for the study extension with the protein vaccine or placebo control (panels C and D). Panels A and C indicate local injection site symptoms; panels B and D indicate systemic symptoms. The percentage of participants who experienced no, mild, moderate, or severe symptoms following each administration of placebo (labeled C), DNA (labeled D), MVA (labeled M), or protein (labeled P) is shown by treatment group for each part of the study.

Forty-eight low-risk participants, 36 in RSA and 12 in the United States, were enrolled over a period of 8 months. Overall, 22 (46%) participants were female, and 37 (77%) were black Africans or African Americans, with a median age of 24 years (range, 18 to 39 years) (Table 3). Forty-four (92%) participants received all 5 vaccinations in the original study (Table 1). Three participants discontinued the vaccination schedule. Twenty-seven participants (6 USA, 21 RSA; 15 male, 12 female) continued in the study extension, with the treatment assignments indicated in Table 2. The vaccine regimen was found to be safe and well tolerated. Most reactogenicity symptoms were graded mild to moderate (Fig. 1). Four people reported severe reactogenicity symptoms: after DNA vaccination, one participant had severe malaise/fatigue; and after MVA vaccination, one person had severe pain at the injection site, one had severe malaise/fatigue, and one reported a severe headache. There were no severe local or systemic reactogenicity symptoms reported after protein vaccination. The 3 pregnancies reported all occurred more than 4 months following the vaccination series and resulted in one full-term live birth, one spontaneous abortion, and one elective abortion.

**TABLE 3:** Demographics and vaccination frequencies

Parameter	Value^*a*^					
	C1 (*n* = 8)	T1 (*n* = 40)	Total (*n* = 48)	C2 (*n* = 10)	T2 (*n* = 17)	Total (*n* = 27)
Sex						
Male	5 (63)	21 (53)	26 (54)	6 (60)	9 (53)	15 (56)
Female	3 (38)	19 (48)	22 (46)	4 (40)	8 (47)	12 (44)
Race						
White	2 (25)	7 (18)	9 (19)	0 (0)	4 (24)	4 (15)
Black/African American	6 (75)	31 (78)	37 (77)	9 (100)	13 (76)	22 (81)
Age (yrs)						
18–20	4 (50)	8 (20)	12 (25)	4 (40)	5 (29)	9 (33)
21–30	2 (25)	25 (62)	27 (56)	3 (30)	9 (53)	12 (44)
31–40	2 (25)	7 (18)	9 (19)	3 (30)	3 (18)	6 (22)
Median	21.5	24.0	24.0	21.0	22.0	22.0
Range	18–35	18–39	18–39	18–37	18–39	18–39
Vaccination frequency						
Day 0	8 (100)	40 (100)	48 (100)			
Day 28	8 (100)	38 (95)	46 (96)			
Day 56	8 (100)	37 (93)	45 (94)			
Day 112	8 (100)	38 (95)	46 (96)			
Day 140	8 (100)	37 (93)	45 (94)			
Extension day 0				10 (100)	17 (100)	27 (100)
Extension day 84				10 (100)	16 (94)	26 (96)

a Data represent numbers (percentages) of participants except where otherwise indicated.

There were no severe or life-threatening adverse events or cardiac adverse events such as myocarditis attributable to the study products. Two participants discontinued vaccinations due to adverse events, one participant because of a schizophrenia relapse after the first DNA vaccination, deemed probably not product related, and the other due to mild right-sided tongue swelling occurring within 90 min of the second MVA vaccination, which resolved spontaneously, deemed possibly related to the study product. Two other participants discontinued vaccinations early: one refused to participate after the first vaccination, and the other was not able to receive the final protein vaccination within the prescribed vaccination window. No participant acquired HIV during the study.

### Immunogenicity. (i) HIV-1-specific T-cell responses.

**FIG 2:**
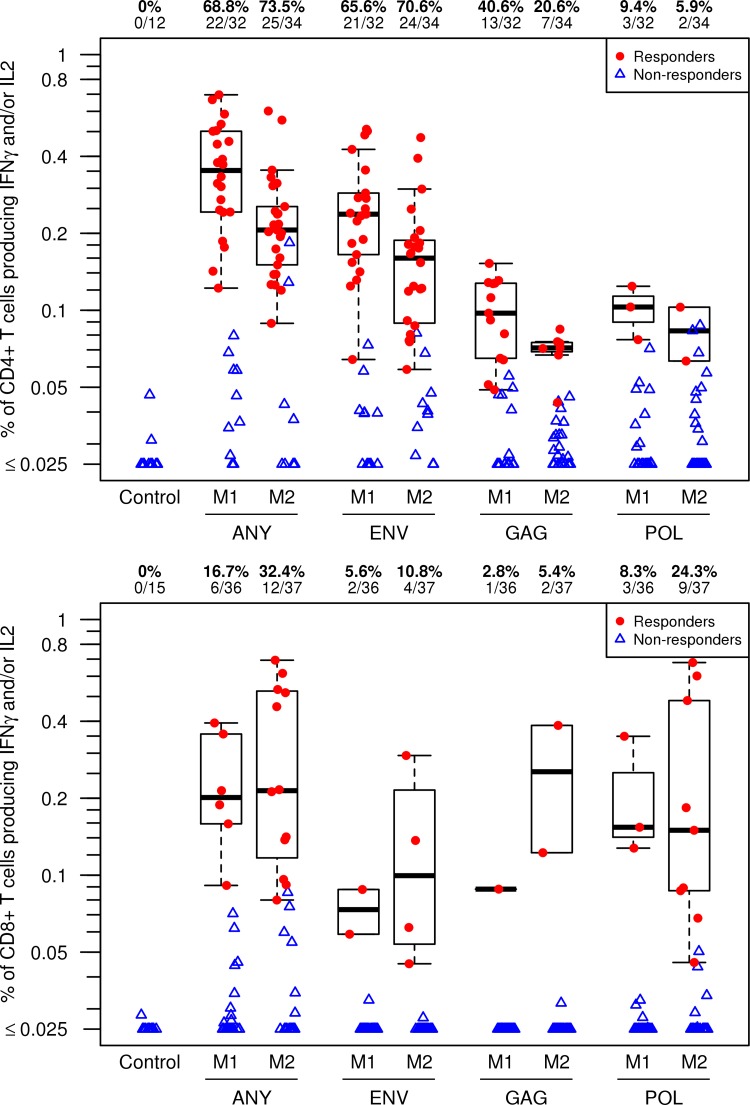
Intracellular cytokine staining assay for vaccine-induced T-cell responses after MVA or placebo vaccinations. The percentages of CD4^+^ T cells (upper panel) and CD8^+^ T cells (lower panel) expressing IL-2 or IFN-γ in response to global PTE peptide pools representing the HIV antigens specifically indicated on the *x* axis or showing a response to any one of these antigens (ANY) are shown. HIV-1-specific CD4^+^ and CD8^+^ T-cell responses were measured using a validated ICS assay. Results are from 2 weeks after the first MVA vaccination (M1) and 2 weeks after the second MVA vaccination (M2). Results from the placebo control group are combined from time points 2 weeks after the fourth and fifth placebo injections (far left). Responder data are shown as red dots, and nonresponder data are shown as blue triangles. Box plots show the distribution of the magnitude of response in positive responders only. The box indicates the median and interquartile range (IQR); whiskers extend to the furthest point within 1.5 times the IQR from the upper or lower quartile. Numbers at the top of each panel show the percentage and number of responders from each group among the evaluable participants.

Vaccine-induced HIV-specific CD4^+^ T-cell responses (i.e., expression of IFN-γ and/or IL-2) to any HIV protein tested were detected in 22/32 (69%) of participants after the first MVA vaccination (Fig. 2, upper panel). While the second MVA boost provided a modest increase in the proportion of individuals with CD4^+^ T cell responses to HIV-1 (to 74%), there was a decrease in the magnitude of the response. For the antigen-specific responses, most participants had CD4^+^ T-cell responses to Env (66%) after the first MVA, which persisted (71%), with a reduced magnitude following the second MVA boost (Fig. 2, upper panel; see also Table S1 in the supplemental material). Fewer responses to Gag were detected (40.6%) after the first MVA and 21% after the second MVA boost), with even fewer participants having CD4^+^ responses to polymerase (Pol) (9.4%).

CD8^+^ T-cell responses were much less frequent than CD4^+^ T-cell responses; 17% of participants had responses to any HIV protein tested after the first MVA, increasing to 32% after the second MVA boost (Fig. 2, lower panel). CD8^+^ T-cell responses were most often seen to Pol (24.3% of participants), with fewer responses to Env (11%) and to Gag (5.4%) after the second MVA boost (Fig. 2, lower panel).

CD4^+^ T-cell responses to any HIV protein after the second MVA boost were detected in 89% of U.S. participants compared to 68% of RSA participants, and CD8^+^ T-cell responses were detected in 44% of the participants in the United States compared to 29% of the participants in the RSA. These differences were not due to differences in cell processing or viability and were not statistically significant (see Table S2 in the supplemental material).

Based on examination of the coexpression results for the 3 cytokines measured, IFN-γ, IL-2, and TNF-α, CD4^+^ vaccine-induced T-cell responses following both MVA vaccination time points were approximately evenly divided among cells producing 1, 2, or 3 cytokines, with slight enrichment for 2 cytokines (data not shown). IL-2 and TNF-α were the dominant cytokines for cells producing one cytokine; coexpression of these was dominant for cells producing 2 cytokines. For CD8^+^ T cells, cells producing 1, 2, or 3 cytokines were detected after the first and second MVA boosts. IFN-γ and TNF-α were the major cytokines expressed, either singly or in combination.

**FIG 3:**
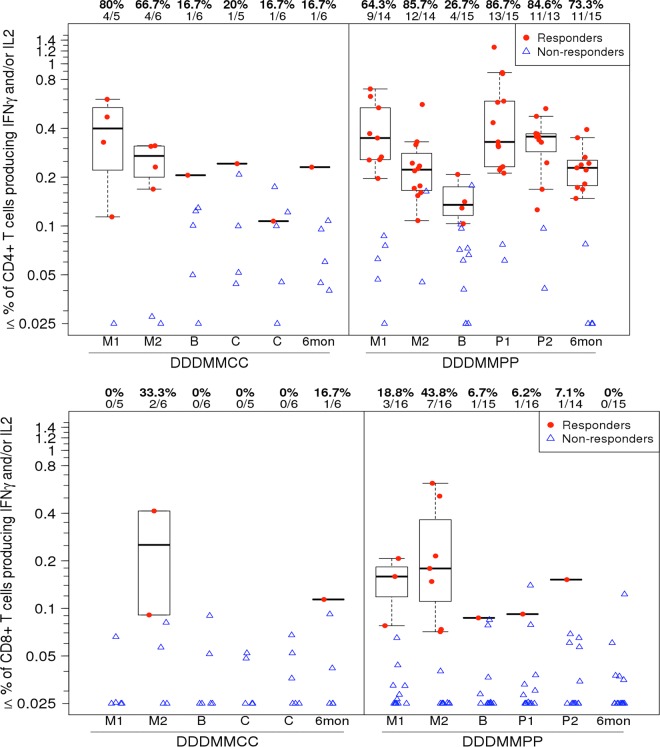
Intracellular cytokine staining assay in study extension participants. The percentages of CD4^+^ T cells (upper panel) and CD8^+^ T cells (lower panel) expressing IL-2 or IFN-γ in response to any antigen represented in the global PTE peptide pools (PTEG) as measured using a validated ICS assay are indicated. Results from study extension participants who had received the DNA/MVA regimen are shown over time, from 2 weeks after the first MVA vaccination (M1), 2 weeks after the second MVA vaccination (M2), at extension baseline (B) prior to injection with gp140 or placebo, 2 weeks after each study extension injection, and 6 months after the final injection. Participants received either placebo (DDDMMCC group) or protein (DDDMMPP). Responder data are shown as red dots, and nonresponder data are shown as blue triangles. Box plots show the distribution of the magnitude of response in positive responders only. The box indicates the median and interquartile range (IQR); whiskers extend to the furthest point within 1.5 times the IQR from the upper or lower quartile. Numbers at the top of each panel show the percentage and number of responders from each group among the evaluable participants.

At the time of the gp140/MF59 protein boost, approximately 2 years following the initial vaccine regimen, HIV-specific CD4^+^ T-cell responses remained detectable in 4/15 (27%) of those participants randomized to receive the gp140/MF59 (DDDMMPP) vaccine, and this proportion increased to 13/15 (87%) of these participants following the first protein boost and was maintained 6 months after the last protein vaccination (73%) (Fig. 3, upper panel; see also Table S1 in the supplemental material). The median magnitude of response following the second protein was similar to the magnitude seen after the first MVA. These response rates after the first and second proteins were significantly different (*P* = 0.014 and 0.0095 [Fisher’s exact test]) from the rates for the participants who received placebo following DNA/MVA vaccination (DDDMMCC), among whom only 20% had responses after the first placebo injection and 17% after the second (Fig. 3, upper panel; see also Table S1). Protein boosting offered no enhancement of CD8^+^ T-cell responses (Fig. 3, lower panel; see also Table S1).

### (ii) HIV-1-specific antibody responses. *(a) Binding antibody responses*.

**FIG 4:**
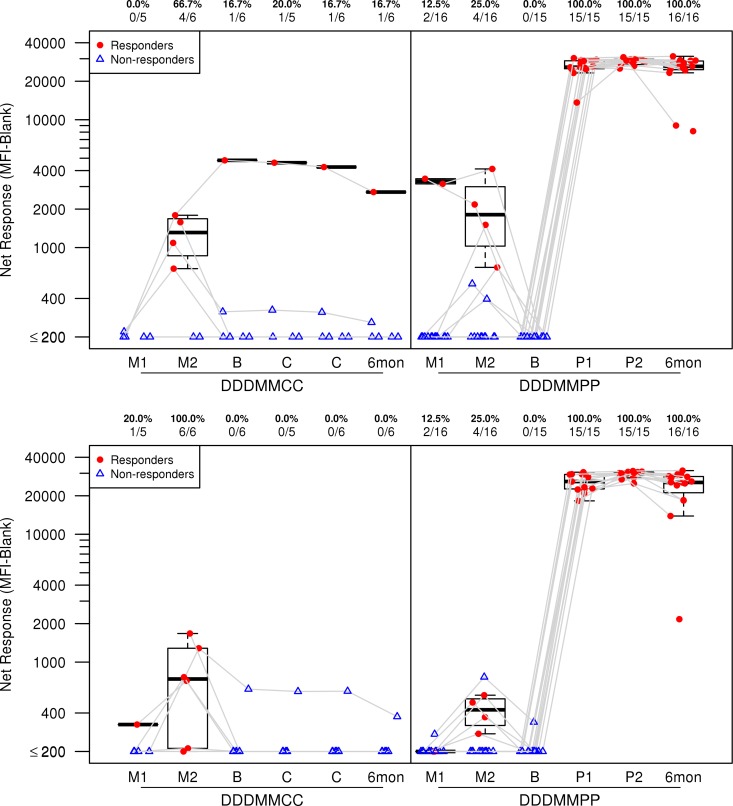
The frequency and magnitude of IgG antibody binding by HIV-1 binding antibody multiplex assay (BAMA). Responses to ConS gp140 CFI (upper panel) and gp140ΔV2.TV1 (lower panel) are shown. The MFI-minus-blank response data summarize the magnitudes at a given time point. Results from study extension participants who had received the DNA/MVA regimen are shown over time, from samples obtained 2 weeks after the first MVA vaccination (M1), 2 weeks after the second MVA vaccination (M2), at extension baseline (B) prior to injection with gp140 or placebo, 2 weeks after each study extension injection, and 6 months after the final injection. Participants received either placebo (DDDMMCC group) or protein (DDDMMPP). Numbers at the top of each panel show the percentage and number of responders from each group among the evaluable participants. Plots include data from responders in red and nonresponders in blue. Box plots show the distribution of the magnitude of response in positive responders only; the midline denotes the median, and the ends of the box denote the 25th and 75th percentiles. Whiskers extend to the most extreme data points within 1.5 times the interquartile range.

DNA/MVA vaccination elicited low levels of HIV-1-specific binding antibodies; the levels were enhanced after boosting with gp140/MF59. At 2 weeks after the second MVA boost, 40.5% (15/37) of the subjects had binding antibody responses to ConS gp140, 67.6% to gp41, and 51.4% to p55 antigen (see Table S3 in the supplemental material). After boosting with gp140/MF59, all (100%) recipients had vaccine-induced binding antibody responses to ConS gp140 (Fig. 4, upper panel [see also Table S3]; also revealed by ELISA results [not shown]), gp41 (see Table S3), and gp140ΔV2.TV1 (Fig. 4, lower panel; see also Table S3) after each protein boost, and the responses were still present 6 months after the final vaccination. The Env IgG titer (AUC) increased after the second protein boost for each vaccine. However, the titers waned in the 6 months following, showing mean declines of 1.8-fold for ConS gp140, 1.4-fold for gp41, and 2.1-fold for gp140ΔV2.TV1 (data not shown). By ELISA, 93% of vaccinees also had responses to the subtype C HIV-1 envelope (Du151.2 gp140), the magnitude of which waned after 6 months (*P* value = 0.0001 [Wilcoxon signed-rank test]). At the initiation of the study extension, 19% of the participants had antibody to p24, and this did not change whether participants were boosted with either protein or placebo. Following the second protein injection, the sole placebo group 1 (C1)/treatment group 2 (T2) participant developed binding antibodies to ConS, gp41, and gp140ΔV2.TV1 (data not shown).

**FIG 5:**
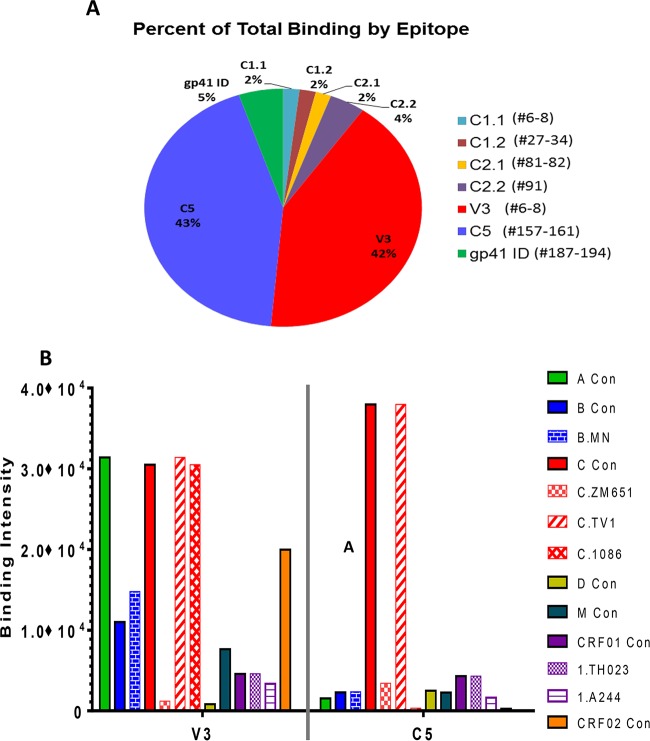
Linear epitope-specific response measured by peptide microarray. (A) Proportions of linear binding responses to each epitope region. The peptide region for each epitope is listed as the peptide numbers in the array library in parentheses next to the name of the epitope. Sequences of all peptides in the microarray library have been published previously (15). For each subject, the percentage values for each epitope are calculated as follows: maximum binding to the epitope/sum of maximum binding to all epitopes. Each pie slice represents the average value for all subjects (*n* = 12) mapped in the study. (B) Clade/strain preferences of the two dominant specificities (V3 and C5). Magnitudes of binding (maximum binding intensities) to V3 and C5 peptides of each clade/strain are plotted. Each bar represents the average value of the results for all 12 subjects mapped.

To evaluate binding specificity, a subset of 12 vaccinees, chosen based on neutralization titers, were examined by IgG linear epitope mapping (Fig. 5). The binding antibodies targeted linear epitopes within the C1, C2, V3, and C5 regions of gp120 and the immunodominant (ID) region of gp41 (Fig. 5A). The dominant responses were against V3 and C5 and were present in 12 of 12 subjects and contributed to 42% and 43% of the overall binding to linear Env epitopes, respectively (Fig. 5A). Notably, IgG responses to the V3 region of the HIV-1 envelope glycoprotein were cross-reactive across multiple HIV subtypes, in contrast to the C5 epitope-specific responses, which were more focused on clade C sequences (Fig. 5B).

### *(b) Neutralizing antibody responses*.

**FIG 6:**
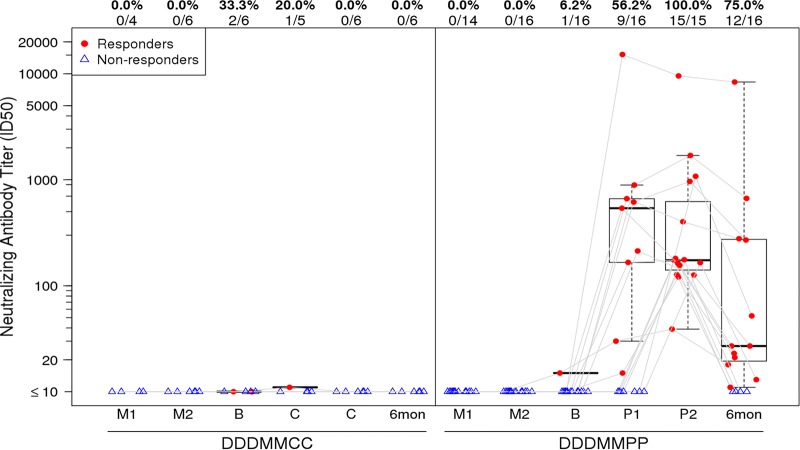
Neutralization 50% tissue culture infective dose (ID_50_) titers in the TZM-bl cell assay against the MW965.26 HIV-1C virus. The titer distributions for each treatment group are shown by time point, from samples obtained 2 weeks after the first MVA vaccination (M1), 2 weeks after the second MVA vaccination (M2), at extension baseline (B) prior to the first injection with gp140 or placebo, 2 weeks after each study extension injection, and 6 months after the final injection. Participants received either placebo (DDDMMCC group) or protein (DDDMMPP). Numbers at the top of each panel show the percentage and number of responders from each group among the evaluable participants. Plots include data from responders in red and nonresponders in blue. Box plots show the distribution of the magnitude of response in positive responders only; the midline denotes the median, and the ends of the box denote the 25th and 75th percentiles. Whiskers extend to the most extreme data points within 1.5 times the interquartile range.

No neutralizing antibodies were detected with the DNA/MVA regimen before the protein boosts. After the first protein boost, neutralizing antibodies to tier 1 viruses MN.3, SF162.LS, and MW965.26 were detected (see Table S4 in the supplemental material). Responses were most frequent against MW965.26, a subtype C virus, and were detected in 56% of participants. After the second protein boost, neutralizing antibody responses to MW965.26 were seen in 100% of the treatment group 1 (T1)/T2 participants and persisted in 75% of those participants for at least 6 months (Fig. 6). No significant responses to the tier 2 viruses, Du151.2 and TV1.21, represented in the vaccines, were seen (see Table S4 in the supplemental material).

## DISCUSSION

Our study results indicate that the DNA/MVA vaccine regimen produced a high frequency of CD4^+^ T cell responses to HIV-1 in healthy non-HIV-infected adults. The protein boost increased the CD4^+^ T-cell response to the HIV-1 envelope and induced binding and neutralizing antibody responses in all participants. At 1.45 × 10^9^ PFU, the dose of MVA used was the highest ever used in a clinical trial. The combination of the three vaccines given in series was generally safe and well tolerated, and the results contribute to the accumulating safety data on DNA/MVA-based vaccines (19, 20, 21) and the TV1 gp140 protein boost with MF59 (22).

The DNA/MVA regimen induced CD4^+^ T cell responses mostly to Env, and the responses persisted in about a quarter of the vaccinated individuals for more than 2 years and were enhanced with the addition of the protein immunogen. Response rates are overall higher than those reported by Goepfert et al. (20) after a similar regimen with two DNA primes followed by two MVA boosts was used and were similar to those seen in RV144, in which 72% of participants had CD4^+^ T cell responses after the last immunization (23). Six months following the protein boosts, the CD4^+^ T cell responses persisted in the majority of participants, although the magnitude slightly decreased. The CD4^+^ T cell responses were measured using PTEG peptides for detection rather than peptides specific for the clade C sequences carried in the DNA/MVA vaccines; the use of clade–matched peptides may have further increased the response rates reported here.

In a recent study, published by the HVTN, that examined the data of 1,218 subjects from 10 phase 1 clinical trials who received only DNA plasmid HIV vaccination, no evidence of tolerance was found (24). The immunogenicity of the DNA plasmid vaccination was influenced by the doses of the DNA, the number of doses received, gender, body mass index, and age. Doses of DNA plasmid HIV vaccines appear to be optimal at the 3-to-6-mg range; thus, we believe that our 4-mg choice delivered an appropriate response. As our DNA prime was followed by a MVA boost, we do not have data on the CD4^+^ T cell and CD8^+^ T cell responses after the DNA prime. Although we did see a reduction in the magnitude of cellular immune responses after the DNA/MVA dosing series, it is difficult to extrapolate the role of DNA in this reduction in the magnitude of response but not in the overall response.

Tier 1 neutralizing antibody responses were present only after the protein boost and were strongest against the HIV-1C isolate. Similarly to findings in other vaccine trials, including RV144, tier 2 isolates were not neutralized by the antibody responses (20). The durability of the neutralizing antibodies was not sustained, declining significantly in the 6 months following the last vaccination, signifying the need for subsequent boosts or other strategies to maintain antibody responses in future trials, if they are found to be correlated with vaccine efficacy.

Binding antibody responses were initially weak after DNA-MVA vaccination. Env binding antibody response rates after the second MVA immunization were not as high those reported by Goepfert et al. (20). However, following the second protein boost, all our participants responded to these antigens and this response was sustained for 6 months after the last vaccination. The protein boost also increased binding antibody titers (magnitude), which were well maintained over 6 months. Goepfert et al. (20) reported a <3-fold decline in the titer magnitude in the first 6 months after vaccination, whereas there was <2-fold decline in our study. The durability of these responses was also considerably better than the 10-fold drop over 26 weeks seen in the RV144 trial (25).

In this study, there were two dominant linear Env epitope specificities, namely, V3 and C5. This is in contrast to RV144, where epitope specificities included C1 and V2, in addition to the V3 and C5 regions of gp120. Levels of IgG to V2 were significantly inversely correlated with infection risk in RV144 (15). However, as the protein in this study had a V2 deletion, antibodies to V2 were not observed after the boost. The response to V3 was also inversely correlated with infection risk in RV144, but only in vaccinees who had lower levels of other antibodies. Responses to C5 showed no significant correlation with infection risk. Interestingly, in HIV-1-infected subjects, dominant responses targeted the V3 and C5 regions of gp120 (15).

Grttn as a polyprotein had relatively low immunogenicity compared to Env, with minimal responses to Nef and no responses to Tat. Env T-cell responses predominated in our study, a result which has also been reported in a number of studies testing poxviruses with multiple HIV gene insertions. CD8^+^ responses to Pol were more frequent than to Gag (24.3% versus 5.4%). Conversely, CD4^+^ Gag responses were more often detected than Pol responses (40.6% versus 9.4% after first MVA). Gag CD4^+^ responses were somewhat lower than Env responses and lower than those reported by Goepfert et al. (20). This may have been due to their use of the Geovax vaccines producing Gag virus-like particles, whereas in this study, Gag was part of a polyprotein which did not bud due to the removal of the myristylation signal (3). T-cell responses to Gag have been correlated with HIV control and may be desirable in an HIV vaccine regimen (26).

In summary, the delayed protein boost enhanced neutralization and CD4^+^ T-cell and binding antibody responses. As with most recombinant pox vector vaccines, CD8^+^ T-cell responses remain limited. Neutralizing antibody responses waned significantly after 6 months, indicating the need for additional boosting or more potent protein boosts. Although binding antibody responses persisted for 6 months, whether this would translate into sustained protection remains to be determined. Evaluating various combinations of different vaccines in prime-boost regimens is necessary in order to design optimal HIV vaccine regimens.

## Supplementary Material

Supplemental material
