# Supplementary material for: Subtype C gp140 Vaccine Boosts Immune Responses Primed by the South African AIDS Vaccine Initiative DNA-C2 and MVA-C HIV Vaccines after More than a 2-Year Gap
Source: Clin Vaccine Immunol. 2016 Jun 6;23(6):496–506. doi: 10.1128/CVI.00717-15 (PMC4895009; doi:10.1128/CVI.00717-15)
Supplement: Supplemental material [file supp_23_6_496__index.html]

Supplemental material 

# Subtype C gp140 Vaccine Boosts Immune Responses Primed by the South African AIDS Vaccine Initiative DNA-C2 and MVA-C HIV Vaccines after More than a 2-Year Gap

## Supplemental material

- Supplemental file 1 -

  Table S1. Proportions of participants with vaccine-induced T-cell responses to PTEG peptides at different timepoints. Table S2. CD4+ and CD8+ T-cell responses, United States and South Africa. Table S3. Frequencies of binding antibody responses via the binding antibody multiplex assay, at major immunogenicity timepoints. Table S4. Frequencies of HIV-specific neutralizing antibody responses via the TZM-bl assay, at major immunogenicity timepoints.

  PDF, 448K
