# Supplementary material for: Subtype C gp140 Vaccine Boosts Immune Responses Primed by the South African AIDS Vaccine Initiative DNA-C2 and MVA-C HIV Vaccines after More than a 2-Year Gap
Source: Clin Vaccine Immunol. 2016 Jun 6;23(6):496–506. doi: 10.1128/CVI.00717-15 (PMC4895009; doi:10.1128/CVI.00717-15)
Supplement: Supplemental material [file CVI.00717-15_zcd999095359so1.pdf]

Supplementary Table 1: The proportion of participants with vaccine induced T-cell responses to PTEG peptides at different timepoints

**Time point**	**Gag**		**Pol**		**Env**		**Any Antigen**	
	CD4	CD8	CD4	CD8	CD4	CD8	CD4	CD8
**HVTN 073 T1**								
**MVA1**	13/32 (40.6%)	1/36 (2.8%)	3/32 (9.4%)	3/36 (8.3%)	21/32 (65.6%)	2/36 (5.6%)	22/32 (68.8%)	6/36 (16.7%)
**MVA2**	7/34 (20.6%)	2/37 (5.4%)	2/34 (5.9%)	9/37 (24.3%)	24/34 (70.6%)	4/37 (10.8%)	25/34 (73.5%)	12/37 (32.4%)
**HVTN 073E T1T2**								
**Extension baseline**	1/15 (6.7%)	0/15	0/1	0/1	4/15 (26.7%)	1/15 (6.7%)	4/15 (26.7%)	1/15 (6.7%)
**Protein1**	0/15	0/16	-	0/1	13/15 (86.7%)	1/16 (6.2%)	13/15 (86.7%)	1/16 (6.3%)
**Protein2**	1/13 (7.7%)	0/14	0/13	0/14	11/13 (84.6%)	1/14 (7.1%)	11/13 (84.6%)	1/14 (7.1%)
**6 Months**	1/15 (6.7%)	0/15	0/15	0/15	11/15 (73.3%)	0/15	11/15 (73.3%)	0/15
**HVTN 073E T1C2**								
**Extension baseline**	0/6	0/6	-	-	1/6 (16.7%)	0/6	1/6 (16.7%)	0/6
**Placebo1**	0/5	0/5	-	-	1/5 (20%)	0/5	1/5 (20%)	0/5
**Placebo2**	0/6	0/6	0/6	0/6	1/6 (16.7%)	0/6	1/6 (16.7%)	0/6
**6 Months**	0/6	0/6	0/6	1/6 (16.7%)	1/6 (16.7%)	0/6	1/6 (16.7%)	1/6 (16.7%)

No responses to Tat and only one response to nef CD4+ response with MVA1 in HVTN 073

Supplementary Table 2: CD4+ and CD8+ T-cell responses, US and South Africa

			Total		USA		RSA		
T-cell	Antigen	Time point	Response Rate	95% CI	Response Rate	95% CI	Response Rate	95% CI	P*
CD4	Any	MVA1	68.8% (22/32)	51.4,82.0	80.0% (8/10)	49.0,94.3	63.6% (14/22)	43.0,80.3	0.44
		MVA2	73.5% (25/34)	56.9,85.4	88.9% (8/9)	56.5,98.0	68.0% (17/25)	48.4,82.8	0.39
	Env	MVA1	65.6% (21/32)	48.3,79.6	70.0% (7/10)	39.7,89.2	63.6% (14/22)	43.0,80.3	1.00
		MVA2	70.6% (24/34)	53.8,83.2	77.8% (7/9)	45.3,93.7	68.0% (17/25)	48.4,82.8	0.69
	Gag	MVA1	40.6% (13/32)	25.5,57.7	60.0% (6/10)	31.3,83.2	31.8% (7/22)	16.4,52.7	0.24
		MVA2	20.6% (7/34)	10.3,36.8	33.3% (3/9)	12.1,64.6	16.0% (4/25)	6.4,34.7	0.35
	Pol	MVA1	9.4% (3/32)	3.2,24.2	0.0% (0/10)	0.0,27.8	13.6% (3/22)	4.7,33.3	0.53
		MVA2	5.9% (2/34)	1.6,19.1	0.0% (0/9)	0.0,29.9	8.0% (2/25)	2.2,25.0	1.00
CD8	Any	MVA1	16.7% (6/36)	7.9,31.9	30.0% (3/10)	10.8,60.3	11.5% (3/26)	4.0,29.0	0.32
		MVA2	32.4% (12/37)	19.6,48.5	44.4% (4/9)	18.9,73.3	28.6% (8/28)	15.3,47.1	0.43
	Env	MVA1	5.6% (2/36)	1.5,18.1	10.0% (1/10)	1.8,40.4	3.8% (1/26)	0.7,18.9	0.48
		MVA2	10.8% (4/37)	4.3,24.7	22.2% (2/9)	6.3,54.7	7.1% (2/28)	2.0,22.6	0.24
	Gag	MVA1	2.8% (1/36)	0.5,14.2	10.0% (1/10)	1.8,40.4	0.0% (0/26)	0.0,12.9	0.28
		MVA2	5.4% (2/37)	1.5,17.7	22.2% (2/9)	6.3,54.7	0.0% (0/28)	0.0,12.1	0.05
	Pol	MVA1	8.3% (3/36)	2.9,21.8	10.0% (1/10)	1.8,40.4	7.7% (2/26)	2.1,24.1	1.00
		MVA2	24.3% (9/37)	13.4,40.1	22.2% (2/9)	6.3,54.7	25.0% (7/28)	12.7,43.4	1.00

Supplementary Table 3: Frequency of Binding Antibody Responses with the Binding Antibody Multiplex Assay, at Major Immunogenicity Timepoints

Antigen	Treatment	Responders at Timepoint % (response rate)		Treatment	Responders at Extension Timepoint % (response rate)			
		MVA 1 or placebo	MVA 2 or placebo		Extension Baseline	Protein 1 or placebo	Protein 2 or placebo	+6 months
**Con S gp140**	T1	10 (1/10)	40.5 (15/37)	T1/T2	0 (0/15)	100 (15/15)	100 (15/15)	100 (16/16)
				T1/C2	16.7 (1/6)	20(1/5)	16.7 (1/6)	16.7 (1/6)
	C1	0 (0/1)	0 (0/8)					
**gp41**	T1	20 (2/10)	67.6 (25/37)	T1/T2	0 (0/15)	100 (15/15)	100 (15/15)	100 (16/16)
				T1/C2	0 (0/6)	0 (0/5)	0 (0/6)	0 (0/6)
	C1	0 (0/1)	0 (0/8)					
**o-gpTV1ΔV2**	T1	ND	ND	T1/T2	0 (0/15)	100 (15/15)	100 (15/15)	100 (16/16)
				T1/C2	0 (0/6)	0 (0/5)	0 (0/6)	0 (0/6)
	C1	ND	ND					
**p24**	T1	10* (2/21)	41* (9/22)	T1/T2	26.7 (4/15)	33.3 (5/15)	20 (3/15)	18.8 (3/16)
				T1/C2	0 (0/6)	0 (0/5)	0 (0/6)	0 (0/6)
	C1	0* (0/5)	0* (0/5)					
**p55 Gag**	T1	30 (3/10)	51.4 (19/37)	T1/T2	ND	ND	ND	ND
				T1/C2	ND	ND	ND	ND
	C1	0 (0/1)	0 (0/8)					

* This was assayed for the subset of participants who rolled over to extension.   Treatment groups combined as T1 = T1/C2+T1/T2 and C1 = C1/C2+C1/T2

Supplementary Table 4: Frequency of HIV-specific Neutralizing Antibody Responses in the TZM-bl Assay, at Major Immunogenicity Timepoints

Isolate	Treatment	Responders at Timepoint % (response rate)			Treatment	Responders at Extension Timepoint % (response rate)			
		Baseline	MVA 1 or Placebo	MVA 2 or Placebo		Extension Baseline	Protein 1 or Placebo	Protein 2 or Placebo	+6 months
Tier 1									
MW965.26 (Clade C)	T1	0 (0/29)	0 (0/32)	0 (0/37)	T1/T2	6.3 (1/16)	56.3 (9/16)	100 (15/15)	75 (12/16)
					T1/C2	33.3 (2/6)	20 (1/5)	0 (0/6)	0 (0/6)
	C1	0 (0/6)	0 (0/6)	0 (0/8)					
MN* (Clade B)	T1	0 (0/29)	0 (0/32)	2.7 (1/37)	T1/T2	0 (0/16)	37.5 (6/16)	60 (9/15)	25 (4/16)
					T1/C2	0 (0/6)	0 (0/5)	0 (0/6)	0 (0/6)
	C1	0 (0/6)	0 (0/6)	12.5 (1/8)					
SF162.LS (Clade B)	T1	ND	ND	0 (0/9)	T1/T2	6.3 (1/16)	43.8 (7/16)	47 (7/15)	25 (4/16)
					T1/C2	0 (0/6)	0 (0/5)	0 (0/6)	0 (0/6)
	C1	ND	ND	0 (0/2)					
Tier 2									
Du151.2 (Clade C)	T1	3.4 (1/29)	0 (0/32)	2.7 (1/37)	T1/T2	0 (0/16)	0 (0/16)	6.7 (1/15)	0 (0/16)
					T1/C2	0 (0/6)	0 (0/5)	0 (0/6)	0 (0/6)
	C1	16.7 (1/6)	16.7 (1/6)	12.5 (1/8)					

TV1.21	T1	ND	ND	ND	T1/T2	0 (0/16)	0 (0/16)	0 (0/15)	0 (0/16)
(Clade C)					T1/C2	0 (0/6)	0 (0/5)	0 (0/6)	0 (0/6)
	C1	ND	ND	ND					

*MN/H9 was tested in the main study, while MN.3 was tested in the extension.

Primary Clade C Tier 2 isolates tested with no responses from T1 or C1: CAP210.2.00.E, CAP45.2.00.G3, Du156.12, Du172.17, Du422.1, ZM197M.PB7, ZM109F.PB4, ZM135M.PL10a, ZM214M.PL15, ZM233M.PB6, ZM249M.PL1, and ZM53M.PB12.

Other Tier 2 isolates tested with no responses from T1 or C1: 92BR025.9, BZ167.12, BaL.26, Bx08.16, SS1196.1.

There were no responses in groups C1/C2 and C1/T2 across visits for all isolates.
